# Psychometric Properties of the Malay Translation of the Medication Adherence Rating Scale

**DOI:** 10.7759/cureus.60570

**Published:** 2024-05-18

**Authors:** Huey Jing Renee Tan, Shiao Ling Ling, Norashikin Khairuddin, Mahmoud Danaee, Arunah Sanggar, Wan Yi Lim, Norliza Bt Chemi

**Affiliations:** 1 Department of Psychiatry and Mental Health, Hospital Kajang, Ministry of Health, Kajang, MYS; 2 Department of Social Preventive Medicine, Faculty of Medicine, University Malaya, Kuala Lumpur, MYS; 3 Department of Psychiatry and Mental Health, Hospital Raja Permaisuri Zainab II, Ministry of Health, Kota Baru, MYS

**Keywords:** medication adherence rating scale, schizoaffective, schizophrenia, compliance, medication adherence

## Abstract

Introduction: The Medication Adherence Rating Scale (MARS) is a self-rated questionnaire that assesses medication compliance.

Aim: This study aimed to validate the Malaysian language translation of the MARS (MARS-M).

Method: The original scale was translated to Malay via forward and backward translation process. The psychometric properties of the MARS-M were validated on clinical samples (N = 54).

Results: The MARS-M was filled by 54 participants. Exploratory factor analysis supported a two-factor model. Factor 1 of the MARS-M consisted of four items (α = 0.84), while factor 2 consisted of three items (α = 0.78).Kaiser-Meyer-Olkin measure of sampling adequacy was 0.60, and Bartlett’s test of sphericity was significant (X^2 ^_(28)_ = 66.4, *p *<0.001).

Conclusion: The MARS-M is reliable and valid.

## Introduction

According to the World Health Report 2001, around 450 million individuals worldwide suffer from psychiatric disorders [1]. In 2019, one in every eight individuals globally was found to be living with a mental disorder [2]. Psychiatric disorders cause substantial global burden and economic burdens. It is projected that the economic burden may increase to about 6.0 trillion American dollars by the year 2030 [3]. Hence, psychiatric disorders appeared to be in the top 20 causes of the global burden of disease (GBD), and it has been estimated that the disease burden for mental illnesses accounts for 32.4% of years lived with disability (YLDs) and 13.0% of disability-adjusted life-years (DALYs). Data showed that mental health conditions in the workplace were estimated to cost the Malaysian economy RM14.46 billion in 2018 [4].

According to the World Health Organization, medication adherence is defined as "the degree to which the person’s behavior corresponds with the agreed recommendations from a health care provider" [5]. Good medication adherence is crucial in the management of patients with psychiatric disorders. However, medication adherence among patients with psychiatric disorders was generally considered rather low. Estimated rates of non-adherence to antipsychotic medication ranged between 25% and 55% in various review studies [6]. Suboptimal medication adherence leads to higher symptom recurrence and is associated with a higher hospitalization rate, increased emergency department consults, and poorer outcomes [6]. Some data showed that medication non-adherence among patients with schizophrenia was estimated to be >50%, which led to higher rates of relapses requiring inpatient treatment and poorer prognosis [7-8].

The World Health Organization identified non-compliance as a worldwide problem of striking magnitude [9]. This problem was not only just found in psychiatric patients but also prevalent in most chronic illnesses [9]. It has been reported that compliance with medications significantly drops after six months of treatment [9]. Medication compliance can be measured via direct and indirect methods. Direct methods involve direct observations and measuring serum levels, whereas indirect methods are pill count, monitoring of prescription refills, and self-report questionnaires [10-11]. Generally, self-report questionnaires are regarded as a common method used in clinical settings as it is easy to use, inexpensive, and not time-consuming. The Drug Adherence Inventory (DAI) and Medication Adherence Rating Scale (MARS) are commonly used in psychiatric research for treatment compliance.

The MARS is a 10-item self-report questionnaire that was developed from two existing scales, which are the Drug Attitudes Inventory (DAI) and Medication Adherence Questionnaire (MAQ) [6]. The MARS has been translated and validated in several different languages, such as German (Cronbach's α 0.60-0.69), Portuguese (Cronbach's α 0.84), Chinese (Cronbach's α 0.83), and Brazilian (Cronbach's α 0.76) [4]. The original MARS was validated in participants with psychoses, including individuals diagnosed with schizophrenia. The MARS is one of the most widely used measurements of adherence in schizophrenia [8]. It has been translated into many other languages, which were also validated in participants with schizophrenia. Moreover, items in the MARS are simple enough to be understood even if patients have thought disturbances. The MARS is a questionnaire used to assess medication compliance, and it is suitable to be used for patients even if they have no insight into their illness. In fact, it is used to understand patients' attitudes toward medication, especially those who lack insights.

Therefore, in this study, the Malay-translated MARS (MARS-M) was validated in a local public hospital setting in individuals with schizophrenia who were in remission and did not have cognitive impairment.

## Materials and methods

Methods

Translation Process

The MARS was translated into Malay via forward and backward translation process. Permission to translate the MARS was obtained from the author. The original MARS was translated from English to Malaysian language by a group of psychiatrists proficient in both English and Malaysian languages to produce MARS-M-I. MARS-M-I was then translated back to English by another group of psychiatrists who are proficient in both languages to produce MARS-MII. MARS-M-II was compared with the original MARS by a team of experts comprising a psychiatrist, psychiatry registrar, and senior medical officer in the psychiatry department. Further editing by the team was done to produce MARS-M-III. Pilot testing for MARS-M-III was done on 30 participants at the psychiatry clinic in a local hospital. Participants were diagnosed with brief psychotic disorder, schizophreniform, schizophrenia, or schizoaffective disorder. To ensure accurate translation of items, the participants were asked about their understanding of the meaning of each item and checked for words or sentences that were confusing or difficult to understand. This was to ensure that the semantic meaning of each item on the scale was retained. This process produced the final version of MARS-M (see Appendix).

Participants and Data Collection Process

The validation study of MARS-M was done on clinical samples in Hospital Kajang, Malaysia. The participants were recruited via systematic randomization. Inclusion criteria were adult patients diagnosed with brief psychotic disorder, schizophreniform, schizophrenia, or schizoaffective disorder. The participants were able to read and understand Bahasa Malaysia. Those who experienced psychotic symptoms or suicidal intent requiring acute intervention and those with neurocognitive disorder or intellectual disability were excluded from the study. Intellectual disability is defined by the diagnostic criteria of the Diagnostic Statistical Manual-5, which is a disorder of both intellectual and adaptive functioning deficits with a childhood onset [12]. All the participants were required to provide consent prior to participation in the study. The participants were required to fill up the sociodemographic form and MARS-M. This study received ethical approval from the Ethics Committee of the National Medical Research Registry, Malaysia (NMRR ID-22-02648-OHA).

Research instrument

MARS-M is a Malay translation of the MARS. The MARS is a reliable and validated scale to estimate compliance with medication in patients with psychoses [10]. It has good internal consistency with Cronbach’s α of 0.75 [10]. It is a self-rated scale consisting of 10 items. The original MARS showed good internal consistency (α = 0.75) with three factors identified, where factor 1 represents "medication adherence behavior" (items 1-4), factor 2 is "attitude toward taking medication" (items 5-8), and factor 3 is "negative side-effects and attitudes to psychotropic medication" (items 9-10) [10]. The participants were required to provide a dichotomous response of either YES or NO for each item. For questions 1 to 6, 9, and 10, a NO response was coded as 1. For questions 7 and 8, a YES response was coded as 1. The sum of scores for all items of ≥9 indicated good compliance. A score of ≥2 and ≤ 8 indicated partial compliance, and a score of 1 indicated poor compliance [10].

Data analysis

Demographic characteristics of the participants were analyzed using descriptive statistics. Exploratory factor analysis was done using the computer program FACTOR [13]. Exploratory factor analysis was employed using a polychoric correlation matrix since data in the study were categorical. Tetrachoric correlation was applied as both variables were dichotomous. Univariate distributions of ordinal items were asymmetric or with excess of kurtosis and polychoric correlation was applied. Factor analysis model for binary variables was used [13]. Principal component analysis (PCA) and promin rotation were used to examine the factor structure of the dichotomous questionnaire items. Parallel analysis was applied to determine the number of factors retained in the scale. Internal consistency was determined via Cronbach’s α.

## Results

Characteristics of the participants

There were a total of 54 participants in the study. The sociodemographic data of the participants in the study are summarized in Table 1. The mean age of the participants was 39.17 (SD = 11.06).

**Table 1:** Demographic characteristics of the participants

Characteristics	N	%
Age (years)		
18–19	1	1.85
20–29	13	24.07
30–39	14	25.93
40–49	12	22.22
50–59	14	25.93
Gender		
Male	25	46.30
Female	29	53.70
Marital status		
Single	27	50.00
Married	20	37.00
Separated	3	5.60
Divorce	3	5.60
Widow/widower	1	1.90
Highest education		
Primary	3	5.60
Secondary	33	61.10
Diploma	7	13.00
Degree	9	16.70
Others	2	3.70
Household income (RM)		
<1000	9	16.7
1000–3999	29	53.7
4000–7999	12	22.2
8000–9999	3	5.6
10000–14999	1	1.9
Occupation		
Professional	4	7.4
Teacher	1	1.9
Businessman	1	1.9
Laborer	2	3.7
Homemaker	11	20.4
Retired	2	3.7
Unemployed	18	33.3
Others	15	27.8

Exploratory factor analysis

Exploratory factor analysis (EFA) was used to determine the factor structure of the 10 items in the MARS. Kaiser-Meyer-Olkin, which was used to measure sampling adequacy, was 0.60. Bartlett’s test of sphericity was significant (X2 (28) = 66.4, p < 0.001). Items 1 and 2 were removed due to the low measure of sampling adequacy (MSA) where values of MSA were below 0.50. Item 6 was removed due to low factor loading. Small values (<0.3) of initial communalities stipulated that variables did not fit well with the factor solution. All initial communalities were above the threshold. All loading factors were noted to be above 0.5 except item 6 (0.091).

The results of EFA on all eight items showed two factors. The eigenvalues and total variance are shown in Table 2. The results after Promin rotation indicated that the first factor (medication adherence behavior) explained 27.1% of the variance, and the second factor (attitudes to taking medication) was 21.4% of the variance. Internal consistency for factor 1 of MARS-M was 0.84, and factor 2 was 0.78. This resulted in a new MARS-M with seven items. The proposed subscale for MARS-M reflected medication-taking behavior (factor 1) and attitude toward medication and subjective experience of side effects (factor 2).

**Table 2:** Factor loadings for the Malay translation of the Medication Adherence Rating Scale

Item	Factor 1	Factor 2
Q3	-	0.718
Q4	-	0.741
Q5	-	0.615
Q7	0.783	-
Q8	0.724	-
Q9	0.696	-
Q10	0.713	-
Eigenvalues	2.218	1.669
% of variance	21.4	27.1

Participant responses and mean score for MARS-M

The participant responses are summarized in Table 3. Following the removal of three items, the total score of MARS-M ranged between 0 and 7. A new cutoff value was calculated for MARS-M based on the ratio of cutoff values in the MARS. A score of ≥6 of MARS-M indicated good compliance, while a score of 1-5 indicated partial compliance and a score of ≤1 indicated poor compliance. The mean score was 5.28 (SD 1.472). The score of the participants is summarized in Table 4.

**Table 3:** Participants' responses to items of the MARS-M MARS-M: Malay-translated Medication Adherence Rating Scale

Item	Participants Response	
	Yes [N (%)]	No [N (%)]
When you feel better, do you sometimes stop taking medication?	14 (25.9%)	40 (74.1%)
Sometimes if you feel worse taking medication, do you stop taking it?	12 (22.2%)	42 (77.8%)
I take medication only when I feel sick.	15 (27.8%)	39 (72.2%)
My thoughts are clearer on medication.	37 (68.5%)	17 (31.5%)
By staying on medication, I can prevent getting sick.	40 (74.1%)	14 (25.9%)
I feel weird, like a "zombie" on medication.	14 (25.9%)	40 (74.1%)
Medication makes me feel tired and sluggish.	5 (9.3%)	49 (90.7%)

**Table 4:** MARS-M scores of the participants MARS-M: Malay-translated Medication Adherence Rating Scale

Items	N (%)
Score ≥6	26 (48.1%)
Score 1–5	27 (50.1%)
Score ≤1	1 (1.9%)

## Discussion

This study presented the development and psychometric evaluation of MARS-M in a clinical setting. MARS-M demonstrated good reliability and validity when applied to individuals diagnosed with schizophrenia. These findings aligned with the psychometric properties observed in the 10-item MARS-10 questionnaire. However, MARS-M exhibited higher internal consistency for factor 1 (r = 0.84) and factor 2 (r = 0.78) in this sample.

Data analysis revealed that MARS-M was valid as a seven-item questionnaire. Items 1, 2, and 6 were removed. It was concluded that these items did not contribute additional information to the analysis, leading to their removal from the questionnaire. Item 1 "Do you ever forget to take medication?" is semantically similar to item 2 "Are you careless at times about taking medication?" Both items 1 and 2 examine the presence of non-compliance to medication intake, which could also be picked up by items 3 and 4 of the questionnaires.

Cultural diversity within Asian communities, as well as individual differences in beliefs and experiences, contributed to a range of perceptions of items in the questionnaire. In Asian cultures, respect for authority figures, including doctors, was often emphasized. There can be shame and embarrassment associated with forgetfulness with medication in some Asian societies, thus fearing judgment or negative consequences [14]. Patients were often hesitant to admit and reveal forgetting medications due to their desire to appear compliant and respectful of medical advice [15]. Thus, they may not answer truthfully to a direct question on whether they have forgotten or were careless in taking medication. Krueger et al. found that self-reported data in clinical settings often overestimated medication adherence by up to 200% [16]. In addition, Lapane et al. indicated a significant discrepancy between doctors' estimates of patients not revealing non-compliance (9%) and patients' actual reluctance to inform their true intentions of not taking the prescription (83%) [17].

In the original MARS, item 6 "It is unnatural for my mind and body to be controlled by medication" is a part of the item assessing attitude to taking medication. However, in MARS-M, results showed that item 6 was neither representative of an assessment of attitude toward medication nor medication side effects. Cultural biases or differences in understanding the concepts of medication as an external and unnatural agent and its effect on the mind and body can contribute to the result. Research has found that Asians have a more negative belief toward medication than Europeans [18-19]. Many Asians believed herbal treatment to be natural, whereas Western psychotropic medications were viewed as unnatural agents that were too strong for their mind and body and may lead to dependence [20].

In addition, individuals with schizophrenia were often found to have a loss of ability to think in abstract concepts leading to concrete thinking [21]. Therefore, individuals with schizophrenia might rely more on concrete thinking rather than integrating information from previous experiences to make judgments and decisions [22]. Some participants were found to have difficulty grasping the concept of natural versus unnatural in item 6 making it difficult to answer item 6.

It was also noted that individuals with a higher education level tend to be more skeptical about psychotropic medications [23]. Patients’ perceptions of health locus of control also influenced their view on the effects of medications [24]. Health locus of control was defined by the degree to which individuals believed that their mental state and body conditions were controlled by internal vs. external factors. [25] Individuals with substance addiction were also more likely to score higher on item 6 compared to individuals with other psychiatric disorders [25]. A large proportion of participants in this study were noted to have dual diagnosis including substance use disorders; however, the actual percentage of participants with dual diagnosis was not collected in this study.

The original MARS had three factors, i.e., medication behavior (factor 1), attitude to taking medication (factor 2), and subjective negative side effects of medication (factor 3) [8,10]. Exploratory factor analysis indicated that MARS-M has two factors. Items 3-4 were grouped as factor 1 and items 7-10 as factor 2. The analysis of MARS-M revealed that items 3 to 5 were of the same construct of a single factor. A multicentered validation study done in France also found that items 3 and 5 were of similar construct [8].

Results from the analysis of MARS-M indicated that items 7-10 were grouped as one factor measuring the same construct. This was different from the original MARS, where items 7 and 8 were measuring attitude to taking medication and items 9-10 were measuring the presence of negative side effects to medication. There were other studies that had similar findings indicating item 7 was close to items 9 and 10 and were grouped as measuring the same construct [8].

The score of MARS-M in this sample showed that half of the participants demonstrated partial compliance to medications, and nearly 50% had good compliance. Generally, medication non-compliance is a significant issue in schizophrenia, with studies reporting compliance rates ranging from as low as 30% to around 60-70% [26-27]. The rate of medication compliance in schizophrenia can vary widely depending on various factors. Factors influencing medication compliance in schizophrenia include the severity of symptoms, side effects of medications, insight into the illness, social support, access to healthcare services, beliefs about medication, and the quality of the therapeutic relationship between patients and healthcare providers [28-29]. Patients’ decisions regarding antipsychotic medication intake also depended on their perception of the overall impact and distress caused by the medication [30]. Therefore, having a rating scale for medication compliance in schizophrenia allows objective measures of medication compliance so that issues with compliance can be identified and addressed effectively.

## Conclusions

Validation studies are crucial for assessing the reliability and validity of questionnaires, such as MARS-M. There were several limitations observed in this study. MARS-M was validated in individuals with psychotic spectrum disorder, i.e., schizophrenia, schizophreniform, brief psychotic disorder, and schizoaffective disorder. This can limit the generalizability of the validation results and restrict the applicability of the questionnaire to disorders other than psychotic disorders. Another consideration is the limitation of a small sample size. In addition, participants of the validation studies may provide responses perceived as socially desirable, rather than reflecting their true experiences or behaviors, which may impact the accuracy of validity estimates.

MARS-M, the Malay adaptation of the MARS, had demonstrated good reliability and validity. Given Malaysia's multicultural makeup and substantial Malay-speaking population, the Malay version of MARS-M serves as a valuable tool for evaluating medication adherence among Malay-literate patients.

## Appendices

Malay-translated Medication Adherence Rating Scale (MARS-M)

**Table 5:** Malay-translated Medication Adherence Rating Scale (MARS-M)

No	Soalan	Jawapan
1	Apabila anda berasa lebih baik, adakah anda kadang-kadang berhenti mengambil ubat anda?	Ya / Tidak
2	Kadang-kadang jika anda berasa lebih teruk apabila mengambil ubat, adakah anda berhenti mengambilnya?	Ya / Tidak
3	Saya mengambil ubat hanya apabila saya sakit.	Ya / Tidak
4	Fikiran saya lebih jelas dengan ubat.	Ya / Tidak
5	Dengan kekal mengambil ubat, saya boleh elak menjadi sakit.	Ya / Tidak
6	Saya berasa aneh, sepoerti zombi apabila mengambil ubat.	Ya / Tidak
7	Ubat membuat saya berasa letih dan lembab.	Ya / Tidak
